# Establishment of Early Endpoints in Mouse Total-Body Irradiation Model

**DOI:** 10.1371/journal.pone.0161079

**Published:** 2016-08-31

**Authors:** Amory Koch, Jatinder Gulani, Gregory King, Kevin Hieber, Mark Chappell, Natalia Ossetrova

**Affiliations:** 1 Veterinary Science Department, Armed Forces Radiobiology Research Institute (AFRRI) Uniformed Services University (USU), Bethesda, Maryland, United States of America; 2 The Henry M. Jackson Foundation, Bethesda, Maryland, United States of America; 3 Scientific Research Department, Armed Forces Radiobiology Research Institute (AFRRI), Uniformed Services University (USU), Bethesda, Maryland, United States of America; Georgetown University, UNITED STATES

## Abstract

Acute radiation sickness (ARS) following exposure to ionizing irradiation is characterized by radiation-induced multiorgan dysfunction/failure that refers to progressive dysfunction of two or more organ systems, the etiological agent being radiation damage to cells and tissues over time. Radiation sensitivity data on humans and animals has made it possible to describe the signs associated with ARS. A mouse model of total-body irradiation (TBI) has previously been developed that represents the likely scenario of exposure in the human population. Herein, we present the Mouse Intervention Scoring System (MISS) developed at the Veterinary Sciences Department (VSD) of the Armed Forces Radiobiology Research Institute (AFRRI) to identify moribund mice and decrease the numbers of mice found dead, which is therefore a more humane refinement to death as the endpoint. Survival rates were compared to changes in body weights and temperatures in the mouse (CD2F1 male) TBI model (6–14 Gy, ^60^Co γ-rays at 0.6 Gy min^-1^), which informed improvements to the Scoring System. Individual tracking of animals via implanted microchips allowed for assessment of criteria based on individuals rather than by group averages. From a total of 132 mice (92 irradiated), 51 mice were euthanized versus only four mice that were found dead (7% of non-survivors). In this case, all four mice were found dead after overnight periods between observations. Weight loss alone was indicative of imminent succumbing to radiation injury, however mice did not always become moribund within 24 hours while having weight loss >30%. Only one survivor had a weight loss of greater than 30%. Temperature significantly dropped only 2–4 days before death/euthanasia in 10 and 14 Gy animals. The score system demonstrates a significant refinement as compared to using subjective assessment of morbidity or death as the endpoint for these survival studies.

## Introduction

The biological effects of radiation on mammalian organisms are strongly dependent upon the radiation dose absorbed. [1] Acute radiation sickness or syndrome (ARS) is classically divided into hematopoietic (HP), gastrointestinal (GI), and neurovascular (NV) sub-syndromes. Each of these sub-syndromes respectively appears in stages directly related to the level of radiation received with some overlap, particularly between HP and GI syndromes. [2, 3]

In humans, HP syndrome occurs after whole-body or significant partial-body irradiation of greater than 1 Gy delivered to the bone marrow. A dose of approximately 3 to 4 Gy may result in death in up to 50% of exposed individuals without significant medical support. [4, 5, 6] Gastrointestinal syndrome and hematopoietic syndrome occur simultaneously at higher radiation doses, beginning at 6 to 8 Gy. Consequences of gastrointestinal syndrome are more immediate and less amenable to treatment. A variety of animal species models have been widely used in prospective research to provide a foundation for the triage and ARS management approaches. [5, 7–22]

Murine radiation models are widely used in radiological countermeasure studies to assess the efficacy of medical countermeasures and to identify and validate candidate biodosimetry assays for radiation dose and injury assessment. [22] Approval by appropriate regulatory agencies for use of medical radiological countermeasure drugs involves demonstration of safety and efficacy, ideally in humans. In cases where human data are inaccessible, relevant animal models should be used. These drugs can be considered effective using the “Animal Rule” wherein two animal species are used instead of unethical testing on humans with potentially lethal or lethal doses of radiation. [12, 14, 23–25] Depending on the endpoint studied, survival studies in rodents typically last 30 days. Although total-body irradiation causes injury in all organ systems, 10 day lethality is generally considered indicative of GI injury, while 30-day lethality generally indicates HP damage. [3, 25]

We previously established animal (*Mus musculus*, *Macaca mulatta*) limited supportive care radiation models to evaluate radiation biomarkers and clinical signs in order to have a predictive model for radiation dose, ranging from 1 to 14 Gy. [7, 8, 13–21]

X- and γ-radiation sources are involved in the majority of accidental radiation exposures. Therefore, the studies were performed using ^60^Co γ-irradiation in the Armed Forces Radiobiology Research Institute’s (AFRRI) ^60^Co facility with reliable and accurate physical dosimetry. The dose rate (0.6 Gy min^-1^) has been established as a standard and well characterized dose rate for animal survival studies at AFRRI and also represents the most reported dose-rate in radiation accidents. [23]

Historically, radiation models have used death as the endpoint in determining efficacy of novel countermeasures or to confirm LD50/30 (the dose at which fifty percent will die within thirty days) as well as other doses, as per the scientific aim. [3, 24,26,27,28] However, allowing an animal to succumb to ARS is not necessary for a statistically valid result. [28] The purpose of this study was to establish criteria, which by tracking animals individually, could significantly and accurately predict imminent death. Variability is introduced both by when animals are checked within observational windows as well as how the course of ARS progresses in individuals, leading to animals potentially being missed as they are not yet ‘morbid’ at check but may be before the next check. With this predictive system, the length of time animals suffer could be significantly reduced through close observation and the better recognition of the signs that lead to morbidity and moribundity. This will greatly reduce the possibility of an animal continuing to live in pain and distress and eventually being found dead.

## Materials and Methods

### Animals

This study was reviewed and approved by the Armed Forces Radiobiology Research Institute (AFRRI) Institutional Animal Care and Use Committee (IACUC). Male CD2F1/Hsd mice (*Mus musculus*, Harlan Laboratories (Indianapolis, IN), www.harlan.com), 12–14 wk old (approximately 22–26 g) were used for these studies. The main objective of these studies was evaluation of biomarkers, dose effects on biomarkers, and agreement with ARS severity scores previously developed. Scoring with Mouse Interventional Scoring System (MISS) was a surrogate for death-as-an-endpoint and was tested concurrent to main investigative aims. Mice were housed 4 per cage under conventional conditions in microisolator filter-top cages in a facility fully-accredited by the Association for Assessment and Accreditation of Laboratory Animal Care (AAALAC) International and treated in accordance with principles outlined in the *Guide for the Care and Use of Laboratory Animals* of the Institute for Laboratory Animal Research, National Research Council. Animal rooms were provided with 10–12 air changes h^-1^ of 100% fresh conditioned air and maintained at 22 (± 2)°C and a relative humidity of 50 (± 20) %. Animals remained on 12:12-h full-spectrum light:dark cycles and received *ad libitum* food (Rodent Diet #8604, Harlan Teklad, Madison, WI) and water (acidified with HCl to a pH of 2.5–2.8). Mice were acclimated 1–2 wk before sham treatment or exposure to ionizing radiation. Clinical observations were conducted twice daily in noncritical and up to four times per day in critical period of ARS (severe pancytopenia), generally 3–14 days post irradiation, though this can vary based on clinical signs and radiation dose. They included temperature readings from microchips (see below) and recording of clinical signs and animal body weight. Animals found to be moribund were euthanized by CO_2_ inhalation followed by cervical dislocation.

### Individual Identification and BMDS Temperature Readings

Mice were identified by IPTT-200, Bio Medic Data System implantable programmable temperature and identification transponders/microchips (BMDS, Seaford, DE) implanted on the mouse back under isoflurane anesthesia, 14 days prior to irradiation. Two weeks allowed for complete recovery after the microchip implantations. Observations were recorded using the Bio Medic Data System electronic data recording.

### Weighing Animals

Since body weight loss is a common clinical manifestation of progressive ARS in humans and animals, body weight was measured daily and at the time of euthanasia in 30-day survival study. Mice were identified and then weighed on a Sartoruis ED5201 scale (Bio Medic Data Systems data acquisition system) and recorded to tenths of grams.

### Exposure of Animals to γ-Radiation

Total-body irradiations (TBI) of mice were carried out in the AFRRI ^60^Co facility. Bilateral irradiation of conscious mice was performed in well-ventilated Plexiglas^®^ boxes. A total of 132 mice were used in this study. Animals were total-body irradiated or treated in the same manner but not exposed to the source (sham-irradiated). The mice were returned to their home cages and the 30-day survival rate was monitored. Control mice were not placed into boxes nor transported to the radiation facility. Comparison of results for sham groups and control groups evaluated any effect of stress induced by handling of mice. Mice in dose cohorts (n = 20 or 26) received TBI at dose rates of 0.6 Gy min^-1^ to the midline over a broad dose range (6–14 Gy). TBI was given as a single exposure. Number of animals per group: control (n = 20), sham (n = 20), 6 (n = 20), 8 (n = 20), 10 (n = 26), and 14 Gy (n = 26) has been determined by the AFRRI statistician from similar studies to provide power > 90% for two-tailed Student’s *t*- tests of less than 10% shifts in value. Dosimetry was performed using an alanine/electron paramagnetic resonance system, with calibration factors traceable to the National Institute of Standards and Technology and confirmed by an additional check against the national standard ^60^Co source of the UK National Physics Laboratory.

### Mouse Intervention Scoring System (MISS)

We define any system of clinical signs given numerical values as a Mouse Interventional Scoring System (MISS). The first MISS (hereafter MISS 1) was developed at AFRRI using scoring systems from other Department of Defense (DoD) and academic institutions as templates and from extensive clinical experience from both veterinary and investigative staff. Certain criteria were selected that were subjectively considered to be signs of morbidity or moribundity in irradiated mice and written into MISS 1. The study MISS (hereafter MISS 2) used for validation is a simplified version of MISS 1. Rigorous staff training was provided to help with uniformity between individuals using the scoring system. A summary of the differences between MISS 1 and 2 is shown in Table 1. Based on the data from MISS 2 as well as clinical and professional observation and judgment, we developed our recommended MISS (MISS 3) shown in Table 2.

**Table 1:** Differences between MISS1 and MISS2.

MISS 1		MISS 2	
**Appearance:**		**Appearance:**	
Normal (smooth coat, clear eyes/nose)	0	Normal	0
Hunched and/or fluffed	1	Reduced Grooming	1
		Dull/rough coat	2
Ocular discharge, and/or edema	3		
		Absence of grooming, piloerection, hunched up	4
Blue mucus membranes/skin (cyanosis)	12		
**Respiratory Rate:**			
Normal breathing	0		
Increased breathing (double normal rate, rapid, shallow)	6		
Abdominal breathing (gasping +/- open mouth breathing)*	12		
**General Behavior:**		**Natural Behavior:**	
Normal (based on baseline observations)	0	Normal	0
		Minor changes, less peer interaction	1
Decreased mobility	2	Little peer interaction, less mobile and alert, isolated	2
		No peer interaction, vocalization, restless or still	4
Ataxia, wobbly, weak**	6		
Inability to stand*	12		
**Provoked Behavior:**		**Provoked Behavior:**	
Normal (moves when cage is disturbed, runs from hand)	0	Normal	0
Subdued; responds to stimulation (moves away briskly)	1	Subdued; responds to stimulation	1
Subdued even to stimulation (moves away slowly)	3	Subdued even to stimulation	2
Unresponsive to gentle prodding **	6	Unresponsive to gentle prodding	4
Does not right when placed gently on side within 5 seconds*	12		
*See* Table 2 *for score grading*		Euthanize if score ≥8	

** Regardless of score, notify appropriate person immediately.

* Regardless of score, immediately euthanize (death is imminent).

**Table 2:** Recommended Mouse Intervention Scoring System (MISS 3).

Appearance:	Normal (smooth coat, clear eyes/nose)	0
	Hunched and/or fluffed	1
	Ocular discharge and/or edema	3
	Pale, white mucus membranes/skin**	6
	Blue mucus membranes/skin (cyanosis)*	12
Respiratory rate:	Normal breathing	0
	Increased breathing (double normal rate, rapid, shallow)	6
	Abdominal breathing (+/-gasping or open mouth breathing)*	12
General behavior	Normal (based in baseline observations)	0
	Decreased mobility	2
	Ataxia, wobbly, weak**	6
	Inability to stand*	12
Provoked behavior	Normal (moves when cage is disturbed, runs from hand)	0
	Subdued; responds to stimulation (moves away briskly)	1
	Subdued even to stimulation (moves away slowly)	3
	Unresponsive to gentle prodding**	6
	Does not right when placed on side within 5 seconds*	12
Weight loss	< 20%	0
	20-25%	3
	26-30%	6
	31-35%	9
	≥35%*	12
**Total Score**		
Score		
< 6	Normal	
6–11	Morbid: Monitor at least 3 times per day; notify appropriate personnel immediately	
≥ 12	Moribund: Notify responsible personnel immediately for euthanasia if no single criterion is 12*. Any single criteria of 12* euthanize immediately; consider as ‘found dead’.	

** Regardless of score, notify appropriate person immediately.

* Regardless of score, immediately euthanize (death is imminent).

Three sets of criteria were selected from MISS 1 to be used in this study and became MISS 2 (study MISS): appearance, general behavior, and provoked behavior. The three criteria each then had four clinical signs described and assigned point values of 0 (normal), 1, 2 and 4. Therefore the total score for MISS 2 is lower than for MISS 1 or 3, with 12 being the highest possible score for MISS 2 whereas it is the value for certain criteria in MISS 1 and 3. MISS 1 and MISS 3 criteria of 6 or 12 point values prompt immediate evaluation for euthanasia; for the study, though these are not part of MISS 2, these signs still prompted consultation with veterinary staff and/or euthanasia.

Appearance was very similar between MISS 2 and MISS 3 except for terminology (e.g. “hunched and/or fluffed” vs. “reduced grooming”) and reflective of the difference in numbering. MISS 2 criteria for appearance also focused on hair coat and were more subtle versus the more specific criteria (e.g. “ocular discharge and edema” in MISS 1 vs. “absence of grooming” in MISS 2) for MISS 3. MISS 1 had criteria which are not found in radiation syndrome except at exposure doses leading to gastrointestinal syndrome. Since this study was not focused on GI syndrome, although at 14 Gy both hematopoietic and GI sub-syndromes occurred, these criteria were not used. For a study looking more specifically at GI syndrome it might be beneficial to add those back in, make a new category, or perhaps replace respiratory signs.

General behavior was the term used in MISS 1 and 3 but was termed “Natural Behavior” in MISS 2. The changes were mostly terminology and point values. MISS 1 and 3 emphasized mobility whereas MISS 2 emphasized peer interaction and mentioned vocalization. Vocalization can be difficult to assess without specialized equipment, however, so is not in the recommended MISS 3. Similarly, peer interaction is impossible to assess in a cage containing only one mouse, which could happen at high doses. When there are multiple animals, our experience is that a mouse becoming affected by radiation will isolate from peers, especially during the day when peers are nesting. This can be helpful screening, but is difficult to quantify. We propose that MISS 3 has easier and more definitive criteria.

Provoked behavior had identical clinical signs; the only change from MISS 1 to MISS 2 was to assign point values of 0–4 instead of 0, 1, 3, and 6 and leaving out the last sign. The last sign in provoked behavior for MISS 1 and 3 was “does not right when placed gently on side within 5 seconds”; medically known as an “absent righting reflex” and a good indicator for immediate euthanasia or intense medical and/or surgical intervention.

In the study, MISS 2 was used and a slightly different cumulative scoring was used. An animal of cumulative score 0 was considered normal, 1–7 in moderate pain or distress and indicating a need for increased monitoring and 8–12 severe and in need of euthanasia. Increased monitoring was done at least twice a day in addition to normal health checks, first check at 6 a.m. and last one at 7 p.m.

### Data Analysis

Statistical analysis was performed using Statistical software (STATISTICA 8—StatSoft, Tulsa, OK). Survival curves were constructed to determine the survival time probability to estimate the LD30/30, LD50/30, and LD70/30. From the daily observational scoring data for each mouse identified using the Mouse Intervention Scoring System (Appearance, Natural Behavior, and Provoked Behavior) a total cumulative score was calculated. Survival time and a percentage of body weight loss were reported in each mouse during the 30-day monitoring after irradiation. The two-sided Student's *t*-test was used when comparing two groups to determine significant difference among groups. Values of P < 0.05 were considered statistically significant. Values were expressed as mean ± standard error (SE).

## Results

The survival study was performed to investigate the biodosimetry endpoints at different doses of radiation (6–14 Gy) on survival of mice. The rationale for the radiation doses selected was based on radiation damage and survival results previously reported in CD2F1 male mice. [3, 25–27] It was demonstrated that mice irradiated to 6 or 8 Gy showed mild or moderate damage and their recovery was certain without or with low risk of critical phase, respectively. In mice irradiated to lethal doses (≥10 Gy), there is severe or fatal damage with a high risk of critical phase and recovery most unlikely due to the HP sub-syndrome in the 10Gy group and a combination of HP and GI sub-syndromes in the 14 Gy group. For this strain of mice, our survival results (LD50 (8.58 [7.97–8.97] Gy) are in a good agreement with earlier published work. [24,25,26]

**Fig 1:**
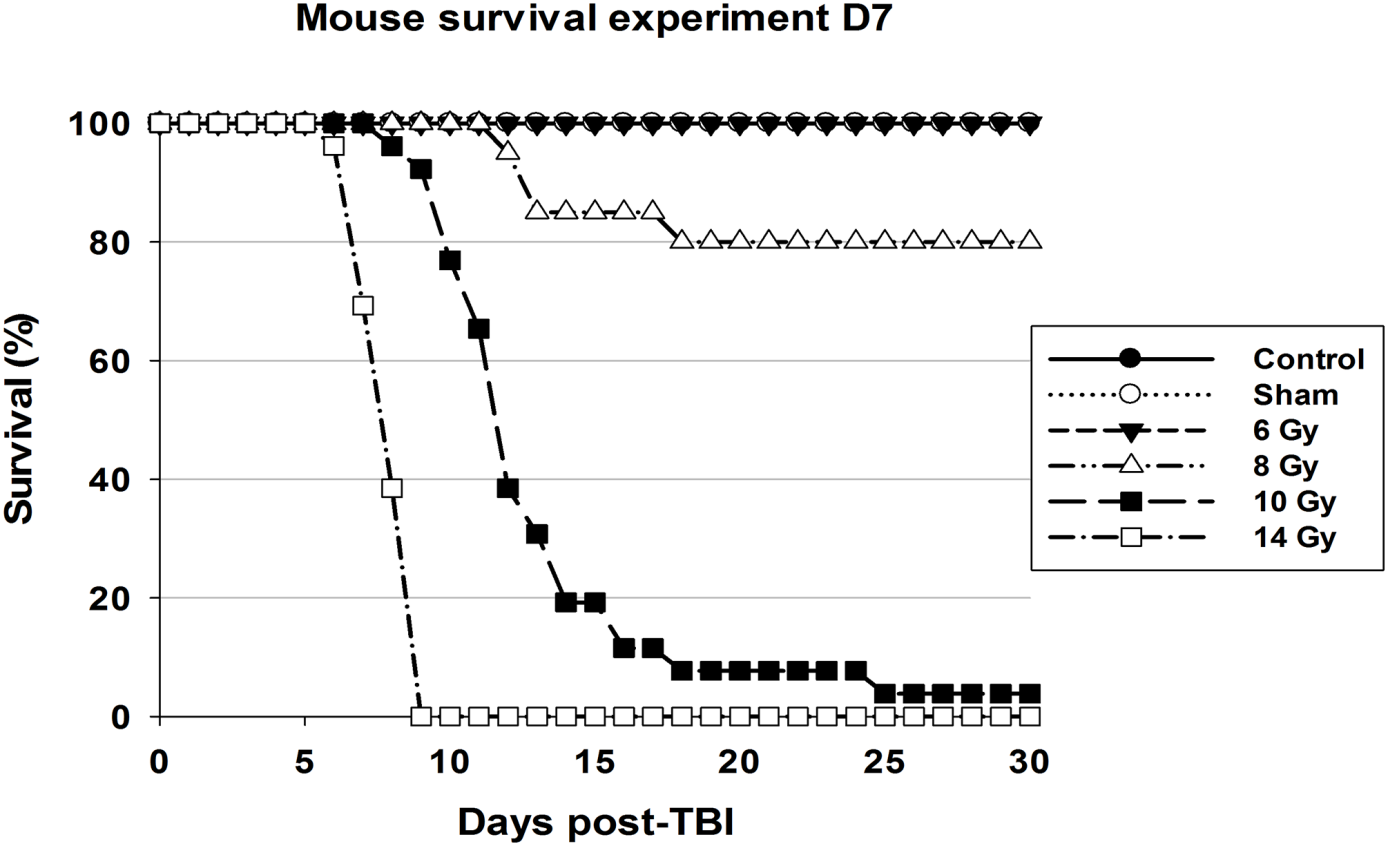
The survival curves for CD2F1 mice: control (•), sham (○) and total-body irradiated to 6 Gy (▼), 8 Gy (Δ), 10 Gy (■), and 14 Gy (□) with ^60^Co γ-rays (0.6 Gy min^-1^). Survival was monitored for 30 days post-TBI; n = 20-26/dose-group.

Furthermore, the objective was to find associations between radiation-responsive biomarkers, body weight, temperature, and signs related to the radiation dose and HP and GI sub-syndromes of the ARS. Animal survival was monitored daily for 30 days after irradiation. Survival curves for CD2F1 male mouse groups: control, sham, and total-body irradiated to 6, 8, 10, and 14 Gy with ^60^Co γ-rays at 0.6 Gy min^-1^ are shown in Fig 1. The survival curves were constructed and probit analysis was performed to determine the 30-day mortality rate. The irradiation doses: LD 30/30, LD50/30, and LD70/30 were estimated as 8.22 Gy, 8.6 Gy, and 8.99 Gy, respectively. From a total of 132 mice (control, sham, 6, 8, 10, and 14 Gy), (92 irradiated), 77 mice survived, 4 mice were found dead in the early morning observations, and 51 mice were euthanized based on established criteria (MISS).

**Fig 2:**
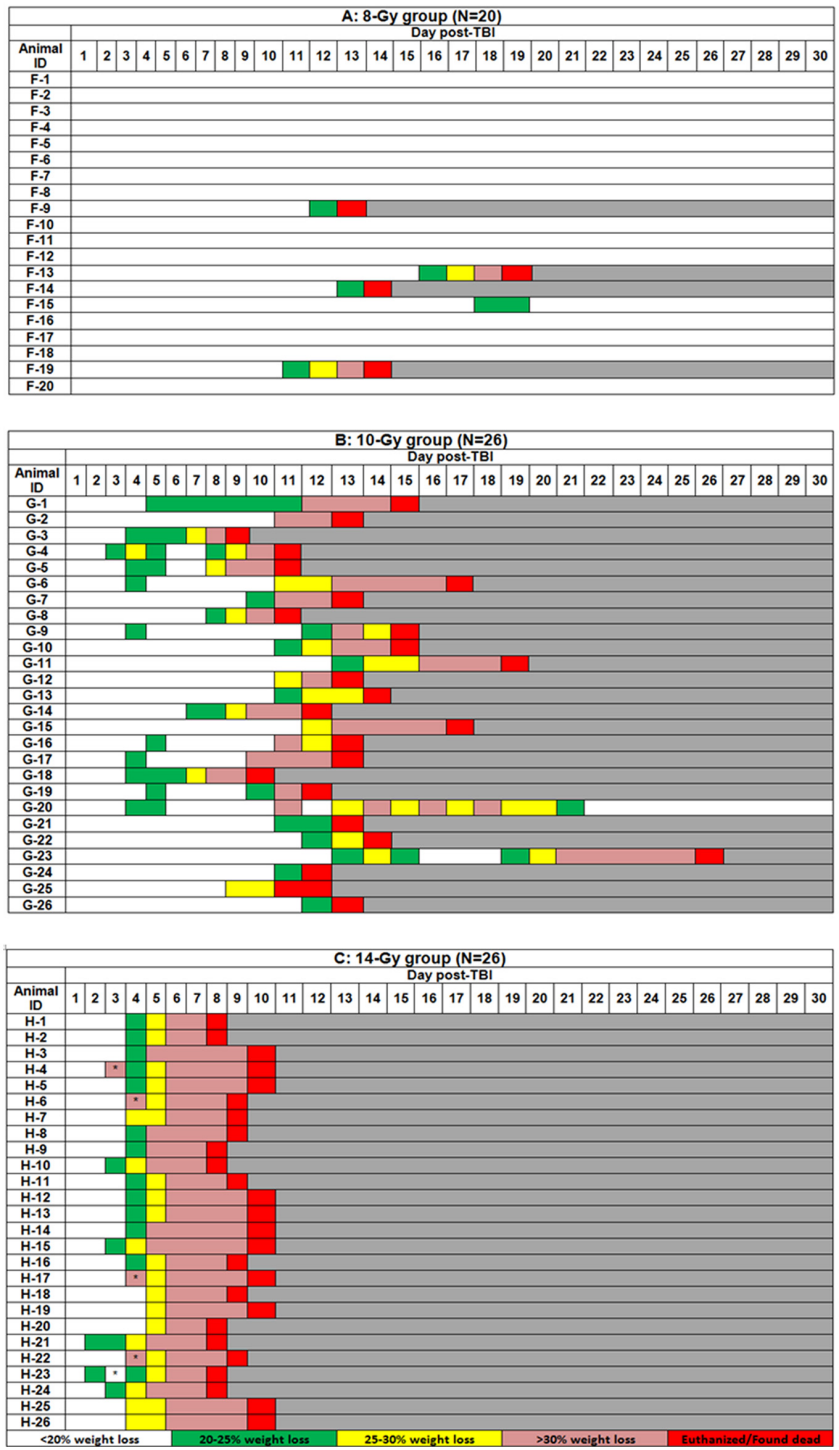
Weight loss percentage in individual mice after total-body irradiation to 8, 10, or 14 Gy with ^60^Co γ-rays (0.6 Gy min^-1^) over a 30-d monitoring period. Mice were observed up to 4 times daily and were humanely euthanized according to the Mouse Intervention Score System criteria (Table 1). Panel A (8 Gy) shows five mice losing over 20% body weight with one of the five mice surviving for the full 30 days. Panel B (10 Gy) shows all mice losing at least 20% (d10 average), 23 losing over 25% (d11 average), and 20 losing over 30% (d12 average) body weight. These mice survived, on average, an additional 5, 2.5 and <1 days respectively after reaching these criteria with one mouse losing over 30% body weight and surviving for the full 30 days. Panel C (14 Gy) shows all mice losing over 30% body weight. On average, mice lost 20% by day 3.5, 25% by d4, and 30% by d6. These mice survived, on average, additional 4.5, 4, and 3 days respectively after reaching these criteria. * Potentially erroneous data due to measurement errors.

Body weight in control and sham mice was in a range (28.4 ± 1.4) g. No significant body weight changes (p<0.306) were observed in control, sham, and 6 Gy groups over the 30 day monitoring period. A significant decrease (p<0.026) was observed in five mice of the 8 Gy group beginning from d10, and in the 10 and 14 Gy groups beginning from d3. Individual time-dependent changes in % body-weight loss are shown in Fig 2. All 6 Gy group mice (n = 20) survived exposure with ≤10% body-weight loss (data not shown). In the 8Gy group (n = 20), a total of five mice lost over 20% of their weight (Fig 2A). Of these five, one survived through 30 days. Four mice lost over 25% of their weight and were euthanized/found dead within a day. Two mice lost over 30% and were euthanized. It was reported earlier that mice irradiated to 8 Gy did not show severe intestinal damage, although 20% were non-survivors due to severe bone marrow damage. [3, 24–27] In the 10Gy group (n = 26), the average day of death was d13.5 (Fig 2B). All mice lost at least 20% of their weight (average d10) and survived an additional 5 days on average. Twenty three mice lost at least 25% (average d11) and survived an additional 2.5 days on average. Twenty mice lost over 30% (average d12) and were euthanized/found dead within a day. One mouse lost over 30% of its body weight and survived the full 30 days (Fig 2B). This mouse was not euthanized as observational criteria were not met (MISS). In the 14Gy group (n = 26), the average day of death was d8 (Fig 2C). All mice lost at least 20% of their weight (average d3.5) and survived an additional 4.5 days on average. Mice losing at least 25% (average d4) survived an additional 4 days. Mice losing over 30% (average d6) survived an additional 3 days on average. Any mice losing over 35% were euthanized within a day.

Appearance scores for mice increased with increasing doses of irradiation. In 6 and 8 Gy animal groups, a dull/rough coat and ocular/nasal discharge was observed in 25% of animals over the entirety of the experiment. In groups irradiated to lethal TBI doses (10 and 14 Gy); additional signs (i.e., absence of grooming, piloerection, and hunching) were observed in all mice (100%). Non-bloody diarrhea was observed only in 14Gy groups beginning from d3-4. Means of the maximum score for appearance in nonlethal and lethal TBI doses displayed over the entirety of the experiment were 2.1 and 2.4, respectively (MISS 2).

**Fig 3:**
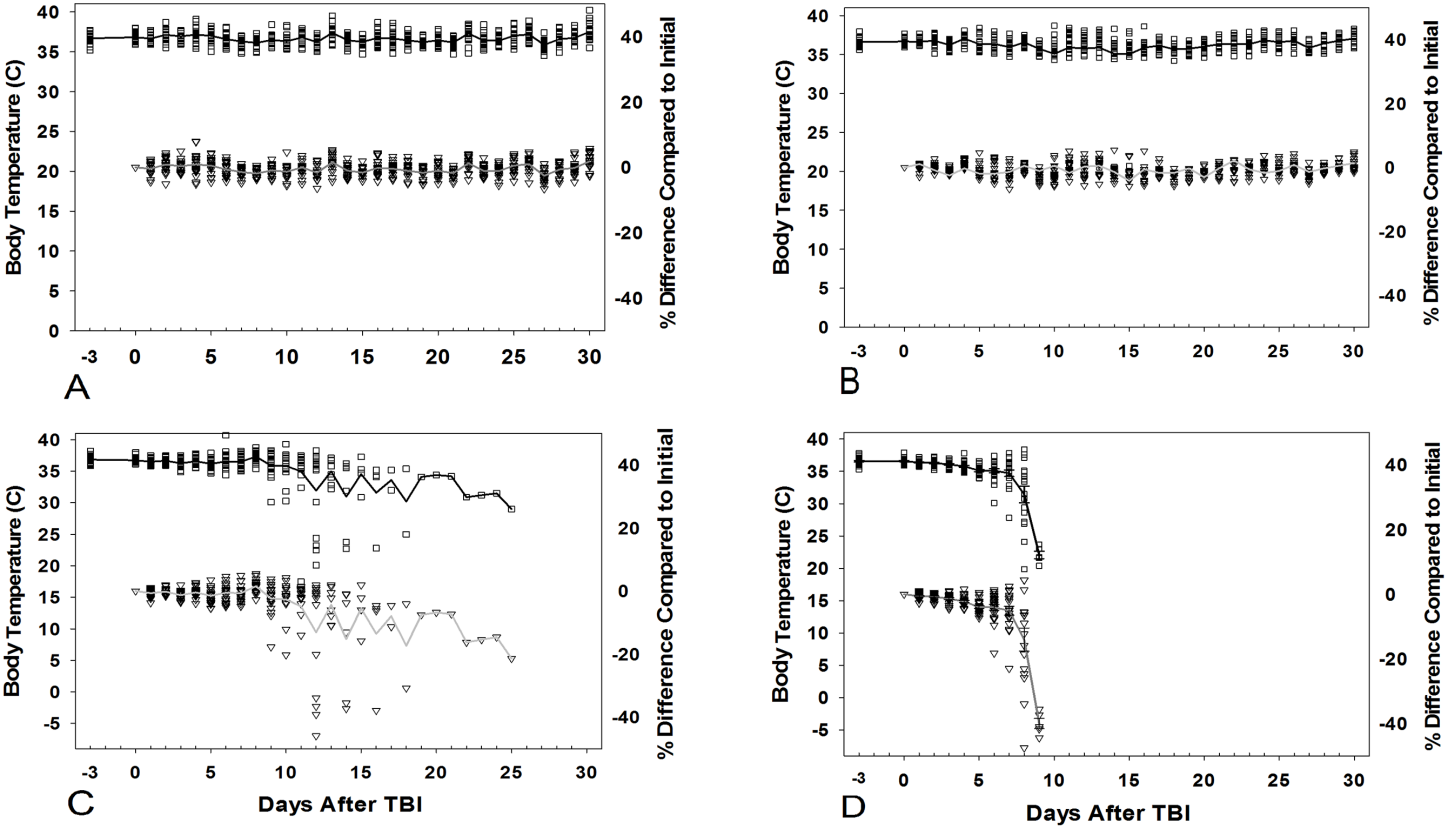
Time-dependent changes in body temperature and percentage difference compared to initial level (-d3) in sham- (A), 8Gy (B), 10Gy (C), and 14-Gy (D) groups of mice over a 30-day monitoring period. The symbols represent individual temperature (**□**) and percentage difference (▽), lines represent the mean values for given number of CD2F1 mice per group and monitoring day.

General behavior of mice irradiated to 6 Gy was similar to those in control and sham groups and was considered normal. Most mice irradiated to 8 Gy showed minor changes and less peer interaction. Four mice (G-9, G-13, G-14, and G-19) expressed more severe signs and were euthanized (Fig 3A). Little peer interaction, less mobility and alertness, and isolation were observed in 10 Gy mice while 14 Gy mice showed no peer interaction or mobility/alertness. Means of the maximum score for general behavior in nonlethal and lethal TBI doses displayed over the entirety of the experiment were 1.5 and 2.5, respectively (MISS 2).

Provoked behavior in mice irradiated to 6 Gy was similar to those in control and sham groups and was considered normal. Some mice irradiated to 8 Gy were subdued but normal when stimulated with the exception of four, which were euthanized (Fig 2A). Two mice irradiated to 10 Gy were subdued even when stimulated beginning from d8-9 and were euthanized the next day, but the majority of them were euthanized after d12 (Fig 2B). Some mice (n = 8) irradiated to 14 Gy displayed unresponsiveness when gently stimulated as well as weakness beginning from d6 and were euthanized the next day (Fig 2C). Means of the maximum score for provoked behavior in nonlethal and lethal TBI doses displayed over the entirety of the experiment were 1.3 and 2.2, respectively (MISS 2).

Means of the maximum total score (all criteria) in nonlethal and lethal TBI doses displayed over the entirety of this experiment was 2.4 and 6.8, respectively, out of a total possible score of 12 and euthanasia cut off score of 8 (MISS 2, Table 1). Therefore lethal TBI doses reflect total scoring on average 3 fold higher than nonlethal TBI doses over the course of the experiment.

Time-dependent changes in body temperature of mice are shown in Fig 3A–3D. Body temperature results in control, sham, and mice surviving exposure to 6 and 8 Gy were in a range (35 ± 3)°C with no significant differences (p<0.277) between groups over the 30 day monitoring period (Fig 3A and 3B). In 10 and 14 Gy animal groups, highly significant (p<0.001) body temperature decreases from 35 (± 3)°C to 28 (± 2)°C (20%) and to 22 (± 4)°C (40%) were observed 2 and 4 days, respectively, prior to death or euthanasia (Fig 3C and 3D).

## Discussion

To our knowledge, this report is the first to successfully refine specific early end point criteria related to ARS studies for a range of doses and types of studies, and is also the most successful to date for identifying animals to be euthanized, with only 7% of non-survivors being found dead. The proposed scoring structure provides a balanced alternative to the use of “death as an endpoint” while ensuring the scientific knowledge sought is balanced with humane use of the animals involved.

A previous study from Nunamaker et al used a predictive cumulative score system to observe and assess the progression of ARS clinical signs over the 30-d monitoring period, which was created to identify humane endpoints in a mouse (C57BL/6, males) TBI model (single radiation dose, LD50 = 8.45 Gy with 6-MV LINAC photon source at dose rate of ~0.8 Gy min^-1^). [14] In that study, mice were weighed only weekly, resulting in a positive predictive value for death of either 80.6% or 84.9% given a 15% or 20% body weight loss, respectively, and was not as strong as it might otherwise have been. Weighing animals every day in our study has increased the predictive value as a humane endpoint. Cumulatively, in our study, from a total of 92 irradiated mice, 51 mice were euthanized versus only 4 mice that were found dead (7% of non-survivors), while in the other study, of total 109 mice irradiated, 25 (~23%) were found dead and 84 (~77%) were euthanized.^17^ In addition to daily monitoring of the body weight loss, the body temperature monitoring allowed the prediction of early death/euthanasia, as highly significant (p<0.001) body temperature decreases from (35 ± 3)°C to (29 ± 6)°C were observed within 1–2 days prior to death or euthanasia.

Definitive criteria were defined as those criteria requiring immediate euthanasia, regardless of observer. These definitive criteria were selected from those criteria which were already being subjectively used by staff to identify animals which would be non-responsive or found dead in the cage within four hours, or roughly the time before the next health check. Other criteria were assigned values based on how likely they could predict, either alone or in combination, acute morbidity or death. Since this was in many respects a subjective assignment, this study allows for adjustment of certain criteria’s point values or cut-offs (e.g. 20% weight loss is likely too strict). Since animals were tracked individually in this study, individuals can serve as their own controls, leading to a refinement in data, for example increased accuracy of weight loss. Often mice are tracked in groups for some or all criteria, either by cage or by some other grouping, thus leading to a potential skewing of some data. Cut-offs for all criteria would need to be adjusted in the event of group tracking. For example in a study where mice are not weighed or not tracked individually, the % weight loss should be stricter to account for averaging if the goal is to euthanize as many mice as possible at humane endpoints.

Weight was not used in MISS 2 (study MISS) as weight was being tracked as part of the study to evaluate the weight loss percentage criteria. Though a common percentage weight loss used as a euthanasia criterion in other studies is 10%, weight loss in radiation from dehydration and other factors was subjectively known to be greater than 10% with a good chance of recovery. The study helped to prove that MISS 1 was too conservative. Most critically, the weight cut-off of 20% loss was shown to be survivable for some mice for many days (see Fig 2), which has also previously been reported^17^. Thus the original cut-off of 20% would have led to premature euthanasia of many mice and skewing of survival curves. MISS 1 and MISS 3 have weight as a definitive criterion. Based on the results of this study additional clinical signs with percentage weight loss were added as a new group of criteria (Table 1 versus Table 2).

Similarly to weight, temperature was noted as a definitive criterion in MISS 1, but was individually tracked in this study and thus not used for MISS 2. Mice were identified by implantable programmable temperature and identification transponders/microchips two weeks prior to beginning the study. This implantable-microchip method reduces the need for intrusive handling of the animals, thereby avoiding additional stress effects.[29] Bio Medic Data System implantable microchips have also been reported being used in ferrets[5, 30, 31] and guinea pigs.[32] Respiratory rate was not evaluated in this study as part of MISS 2 but can be helpful. This measure was one of the ones used when evaluating mice which were equivocal (a mouse of cumulative score 7 as per MISS 2) and was helpful in determining how soon the increased monitoring was needed. In an animal, for example, of cumulative score 7 with labored breathing a recheck would be done in one half hour versus another of cumulative score 7 which was breathing normally. Especially at higher doses of irradiation the change in cumulative score from under a euthanasia cut-off to moribund can take place in very short timeframes.

Body temperature results demonstrated that in control, sham, and mice surviving exposure to 6 and 8 Gy, no significant differences were observed among those groups over the 30-d monitoring period. In 10 and 14Gy animal groups, highly significant temperature decreases were observed one or two days prior to humane euthanasia based on criteria presented in MISS 2. Temperature proves a very sensitive measure as a humane endpoint; however catching it in time can be problematic if there are longer periods between assessing this parameter, for example, at night. Additionally though animals were individually tracked as part of this study with implantable microchips reading temperatures, more analysis would be needed to find cut-offs. Additionally most mice are not microchipped in radiation studies. [3, 24–26] A better way to get accurate temperatures that is, or at least changes in temperatures, less invasive for mice would be necessary to use this cut-off broadly. MISS 1 had ‘cool to the touch’ as a definitive criteria but proved too subjective to use reliably. It was thus taken out of both the MISS 2 and MISS 3.

Based on the results of this study, MISS 2 was changed with the data generated in this article to inform MISS 3 (Table 2). We propose that MISS 3 be used for studies of ARS in mice. Other studies have used systems similar to ours effectively, but did not have individual tracking as in this study so it is hard to determine statistically how well particular criteria worked. [2, 14] Criteria selected were closely correlated with morbidity and the goal was met of reducing ‘death as endpoint’ by incorporating euthanasia criteria already in use at AFRRI based on scientific and veterinary experience. This constitutes a refinement of animal use in radiation studies. Subsequent to this study, data are now available to either confirm or cause adjustments to the original score sheet (MISS 1) to current score sheet (MISS 3), which is considered a living document at this time. Further studies are needed to statistically evaluate all criteria; best wording for “appearance”, respiratory rate, wording and point values for “General behavior” compared to actual mortality as well as point totals. As it is analyzed and improved, such a score sheet can be used in future studies to achieve the lowest possible found dead versus euthanized. Due to the nature of ARS, it is unlikely that spontaneous death can be entirely avoided, but coming to an understanding of the physiologic parameters which precede death will not only refine animal use but increase understanding of injury caused by ionizing radiation.
